# Formation of a photocatalytic WO_3_ surface layer on electrodeposited Al–W alloy coatings by selective dissolution and heat treatment

**DOI:** 10.1038/s41598-019-52178-6

**Published:** 2019-11-05

**Authors:** Shota Higashino, Masao Miyake, Takumi Ikenoue, Tetsuji Hirato

**Affiliations:** Graduate School of Energy Science, Kyoto University, Yoshida-honmachi, Sakyo-ku, Kyoto 606-8501 Japan

**Keywords:** Corrosion, Photocatalysis

## Abstract

In this study, we explored the feasibility of WO_3_ surface layer formation on electrodeposited Al–W alloy coatings by selective dissolution and heat treatment, with the aim of providing corrosion-resistant Al–W alloy coatings with photocatalytic self-cleaning properties under visible light illumination. The selective dissolution of Al and oxidation of residual W was carried out by immersing Al–W alloy films in an aqueous solution of nitric acid. A nanostructured H_2_WO_4_·H_2_O surface layer was formed on the alloy film by this process. The H_2_WO_4_·H_2_O layer was dehydrated to WO_3_ by heat treatment, yielding a multilayered WO_3_/Al–W alloy film with an approximately 300 nm thick WO_3_ layer. The WO_3_/Al–W alloy film exhibited photocatalytic self-cleaning, as demonstrated by the photodegradation of stearic acid and methylene blue. We also confirmed that selective dissolution and heat treatment did not significantly diminish the corrosion resistance of the Al–W alloy films.

## Introduction

Aluminum and its alloys are highly resistant to corrosion and oxidation. They have consequently attracted attention as corrosion-protective coatings for reactive materials, such as Mg alloys and steels^1–3^. Among aluminum-based binary alloys, Al–W alloys are known to have the highest resistance to chloride-induced pitting corrosion^4,5^. Al–W alloys have been formed using sputtering^4–11^, ion implantation^12^, laser alloying^13^, and electrodeposition^14–19^. Electrodeposition is advantageous for industrial applications because a thick film can be formed rapidly on a large substrate using uncomplicated equipment. Recently, we reported that dense Al–W alloy films with W contents of up to 18 at.% could be electrodeposited from 1-ethyl-3-methylimidazolium chloride (EMIC)–aluminum chloride (AlCl_3_) ionic liquids containing tungsten(II) chloride (W_6_Cl_12_)^17–19^.

Self-cleaning coatings have been developed extensively, owing to the practical advantages of energy savings and environmental compatibility^20–23^. Such coatings can be obtained by forming a hydrophilic surface layer with a photocatalytic material, such as titanium dioxide (TiO_2_), which catalyzes the photodecomposition of adsorbed organic compounds^20,21,23–26^. TiO_2_-based coatings only exhibit self-cleaning properties when exposed to UV illumination, such as sunlight. This behavior is due to the wide bandgap of TiO_2_ (~3.2 eV). In contrast, tungsten oxide (WO_3_), which has a narrower bandgap of 2.5–2.8 eV, can absorb visible light energy. WO_3_ thus exhibits self-cleaning properties under visible light illumination^27–30^.

Herein, we describe a new process for imparting corrosion-resistant Al–W alloy films with self-cleaning properties through the formation of a WO_3_ surface layer. This process is comprised of a chemical dissolution step and heat treatment. Al is selectively removed by dissolution in an acidic solution, and a W-enriched layer is formed at the surface of the Al–W alloy film. Subsequent heat treatment in air converts the W-enriched surface layer to photocatalytic WO_3_. Through this process, a corrosion-resistant Al–W coating with self-cleaning ability under visible light illumination is obtained. Many strategies for preparing WO_3_ surface layers have been reported in the literature, including sputtering^31–33^, chemical vapor deposition^34^, vacuum evaporation^35^, spin-coating^27^, spray pyrolysis^36,37^, sol–gel processing^38,39^, and hydrothermal growth^28,40^. The process described herein differs from these routes in that W present in the alloy film itself serves as the WO_3_ precursor; hence, no additional W precursor is required. For this reason, this process is cost-effective and facilitates successive and large-scale production.

In this study, we examined the feasibility of forming photocatalytic WO_3_ layers on Al–W alloy films via this process. The conditions required for the formation of photocatalytic WO_3_ on Al–W alloy films were determined. Then, the self-cleaning properties of the WO_3_/Al–W alloy films were evaluated by monitoring the photodegradation of stearic acid (SA) and methylene blue (MB) under visible light illumination. Finally, the corrosion resistance of the WO_3_/Al–W alloy films was confirmed.

## Results and Discussion

### Electrodeposition of Al–W alloy films

**Figure 1:**
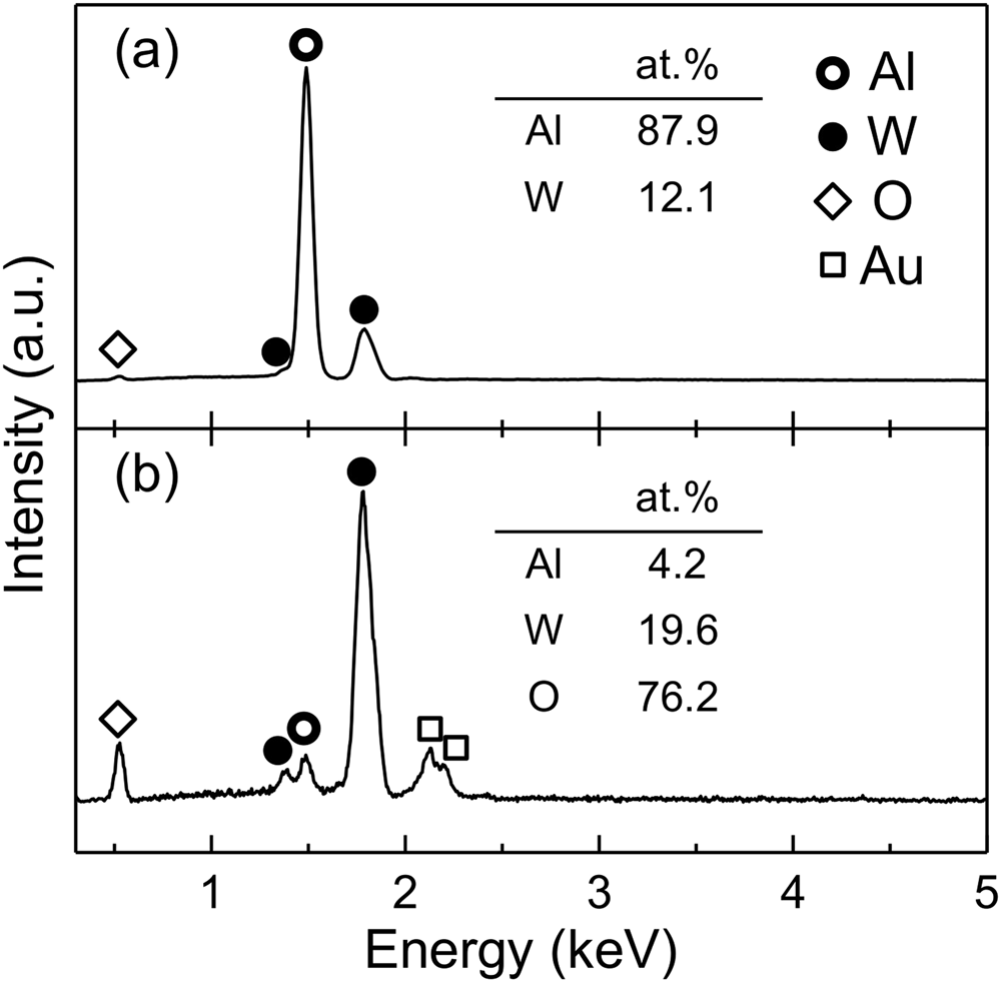
EDX spectra of (**a**) as-deposited Al–W alloy film and (**b**) Al–W alloy film immersed in aq. HNO_3_ for 15 h. The Au in (**b**) is due to the sputtered Au coating added to prevent charging effects.

**Figure 2:**
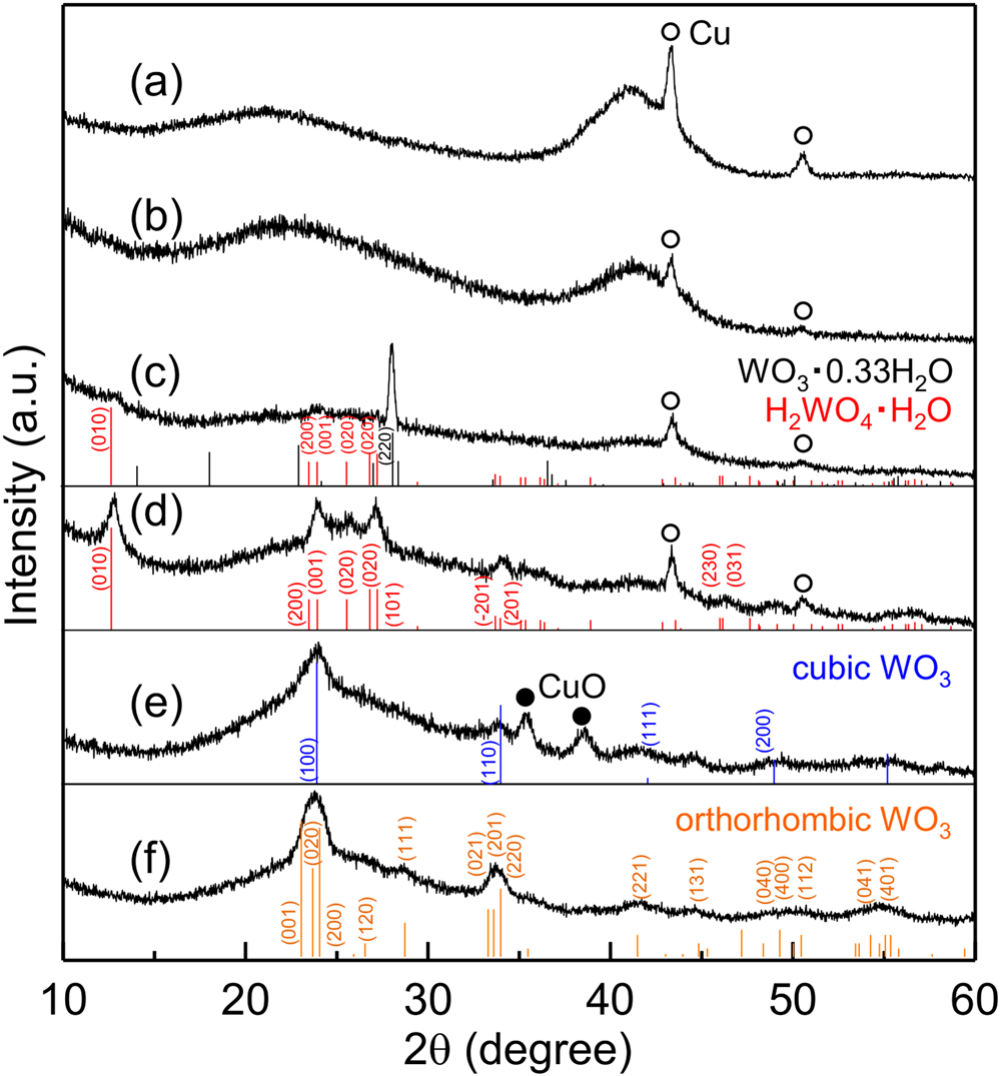
XRD patterns of (**a**) as-deposited Al–W alloy films and the films after immersion in aq. HNO_3_ for (**b**) 9 h, (**c**) 12 h, and (**d**) 15 h. XRD patterns of the films after heat treatment at (**e**) 300 °C and (**f**) 350 °C. The diffraction patterns for Cu and CuO are derived from the Cu substrate outside the electrodeposition area and its oxidation layer. The reported peak positions for WO_3_·0.33H_2_O (JCPDS: 01-087-1203, *Fmm2*, *Z* = 12; *a* = 0.73447 nm, *b* = 1.25470 nm, *c* = 0.77367 nm), H_2_WO_4_·H_2_O (JCPDS: 18-1420, *P2/m*, *Z* = 2; *a* = 0.75000 nm, *b* = 0.69400 nm, *c* = 0.37000 nm, *β* = 90.5000°), cubic WO_3_ (JCPDS: 41-0905, $Pm\bar{3}m$, *Z* = 1, *a* = *b* = *c* = 0.37140 nm), and orthorhombic WO_3_ (JCPDS: 20-1324, *Pcnb*, *Z* = 4, *a* = 0.73840 nm, *b* = 0.75120 nm, *c* = 0.38460 nm) are also shown.

The typical energy-dispersive X-ray spectroscopy (EDX) spectrum of an electrodeposited Al–W alloy film in Fig. 1a indicates that only Al and W are present in the film. No other elements were detected, except for a small amount of O due to surface oxidation. The W content of the alloy film was determined to be ~12 at.% based on the EDX spectrum. The X-ray diffraction (XRD) pattern (Fig. 2a) shows halos located at approximately 2θ = 21° and 42°, indicating that the as-deposited alloy film was amorphous. The average film thickness was ~11 μm.

### Selective dissolution of Al–W alloy films

In the first attempt to form a surface oxidation layer, the electrodeposited Al–W alloy film was heated in air. However, thermal oxidation of the as-deposited film generated a complex oxide (Al_2_O_3_·3WO_3_) rather than photocatalytically active WO_3_ (Supplementary Fig. S1). The photocatalytic activity of the thermally oxidized film (Al_2_O_3_·3WO_3_/Al–W alloy) was negligible (Supplementary Fig. S2).

To obtain WO_3_ instead of the complex oxide, the W content of the alloy film had to be increased prior to thermal oxidation. The Al–W alloy films were immersed in 0.56 M (3.5 wt.%) aqueous nitric acid (HNO_3_) solution before heat treatment, with the intention of dissolving Al selectively to form a W-enriched surface layer. Figure 1b shows a typical EDX spectrum collected from the surface of an alloy film immersed in aqueous HNO_3_ for >15 h. The amount of Al was significantly reduced after immersion in aq. HNO_3_, indicating that Al was selectively dissolved from the Al–W alloy films. This result is consistent with the expected behavior based on potential–pH diagrams^41^, which show that Al dissolves in acidic solutions, whereas W is passivated. The increased amount of O indicated that oxidation of residual W proceeded simultaneously during the dissolution process. The XRD patterns of the films after selective dissolution for different durations are shown in Fig. 2b–d. The pattern of the film after 9 h (Fig. 2b) contained no diffraction peaks except for the amorphous halo pattern of the Al–W alloy. In contrast, faint diffraction peaks attributed to H_2_WO_4_·H_2_O (JCPDS: 18–1420) and an intense peak near 28° were observed in the pattern of the film treated for 12 h (Fig. 2c). The peak near 28° may be derived from WO_3_·0.33H_2_O, but this is not a certainty owing to the absence of other diffraction peaks. The film treated for >15 h (Fig. 2d) clearly yielded a diffraction pattern corresponding to H_2_WO_4_·H_2_O.

Liu *et al*. reported that selective dissolution of a sputtered W-rich Al–W alloy film (>45 at.% W) under similar conditions yielded a metallic β-W phase^42^. The formation of H_2_WO_4_·H_2_O in the present work could be attributed to the lower W content of the starting alloy, which contained 12 at.% W. The dissolution of Al from the Al–W alloy with the lower W content should generate a large number of atomic vacancies and the residual W atoms should have relatively weak metallic bonding to each other. Thus, these W atoms are highly active, resulting in a higher probability of being oxidized to form bulk H_2_WO_4_·H_2_O^43,44^. The formation of H_2_WO_4_·H_2_O by selective dissolution of electrodeposited Fe–W alloy films has also been reported, although the W content of the alloy films was not indicated^45^.

**Figure 3:**
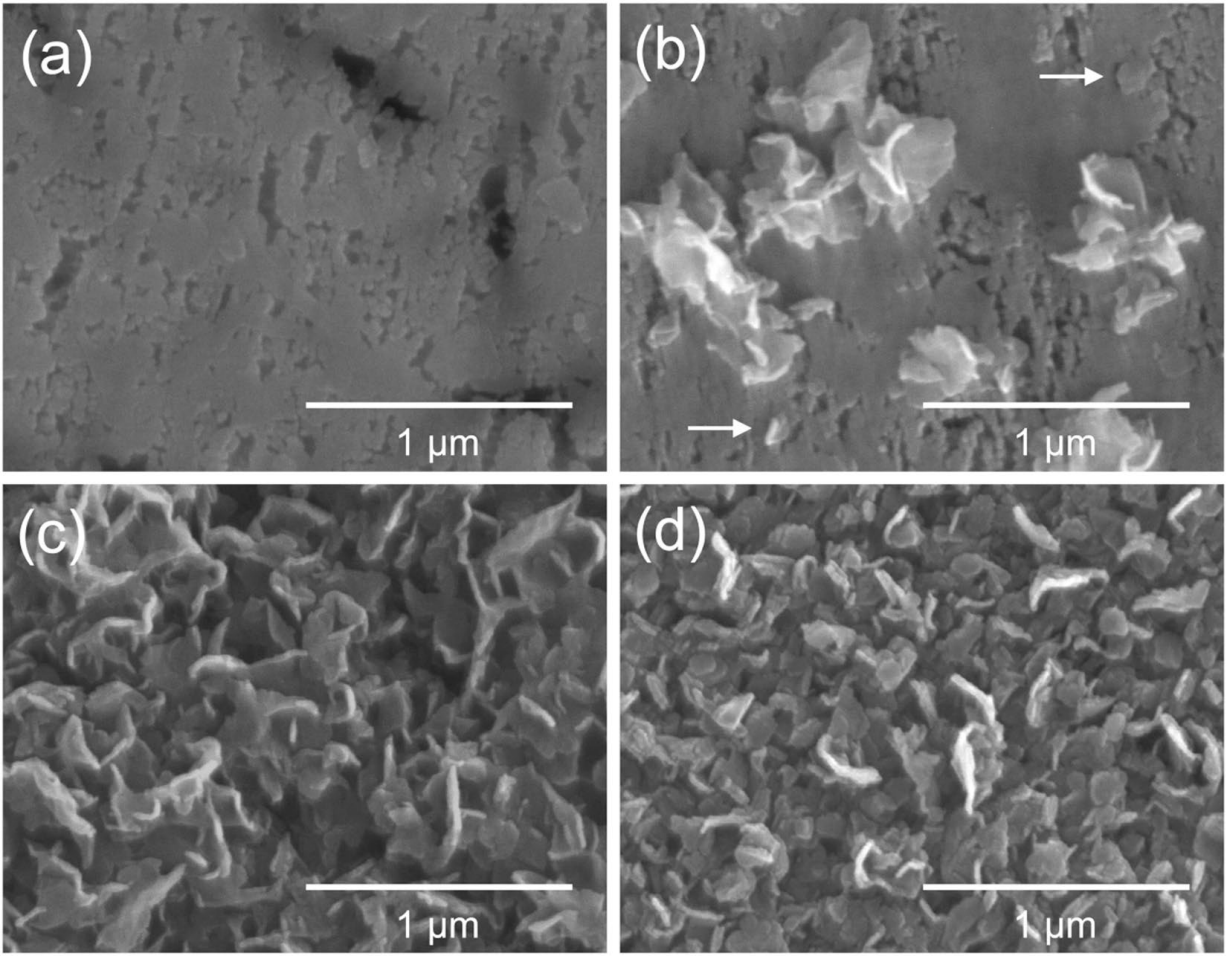
Surface SEM images of Al–W alloy films after selective dissolution for (**a**) 9 h, (**b**) 12 h, (**c**) 15 h, and (**d**) 24 h. The arrows in (**b**) indicate exfoliation of the film along the cracks with one side remaining attached to the film surface.

Figure 3 shows surface scanning electron microscopy (SEM) images of the alloy films after selective dissolution for various durations. Small cracks a few hundred nanometers in length were observed at 9 h (Fig. 3a). These cracks were attributed to volume shrinkage caused by Al dissolution at the surface. With increasing duration, portions of the film along the cracks were exfoliated, but one side remained attached to the film surface to form petal-like structures, as indicated by arrows in Fig. 3b. Based on the XRD pattern shown in Fig. 2c, the petals in Fig. 3b were composed of WO_3_·0.33H_2_O and H_2_WO_4_·H_2_O. The petals covered the entire surface by 15 h (Fig. 3c), at which point they were composed of H_2_WO_4_·H_2_O (Fig. 2d). The width and thickness of each H_2_WO_4_·H_2_O petal were <300 nm and <50 nm, respectively. A similar petal-like morphology is commonly observed following selective dissolution of sputtered Al–W alloy films containing >45 at.% W^42^ and electrodeposited Fe–W alloy films^45^, although the residue of the former is composed of β-W and that of the latter is composed of H_2_WO_4_·H_2_O. Increasing the dissolution duration from 15 to 24 h (Fig. 3c,d) did not cause a notable change in the petal-like morphology, but the film treated for 24 h suffered from significant volume shrinkage that generated macroscopic cracks a few micrometers in width. Based on these results, a dissolution duration of 15 h was selected for the following experiments.

### Dehydration of H_2_WO_4_·H_2_O to WO_3_

Since H_2_WO_4_·H_2_O is known to have negligible photocatalytic activity^46,47^, heat treatment was performed in air to dehydrate H_2_WO_4_·H_2_O to photocatalytic WO_3_. The XRD patterns obtained after heat treatment (Fig. 2e,f) show that H_2_WO_4_·H_2_O was converted to cubic (c)-WO_3_ (JCPDS: 41-0905) at 300 °C and orthorhombic (o)-WO_3_ (JCPDS: 20-1324) at 350 °C. These results were consistent with those in a previous report^48^, which showed that H_2_WO_4_·H_2_O was dehydrated to c-WO_3_ at 300 °C and converted to o-WO_3_ by heating at 300 °C for a longer duration. The X-ray photoelectron spectroscopy (XPS) W 4 f spectra of the films showed that W existed as W^4+^, W^5+^, and W^6+^ in H_2_WO_4_·H_2_O, with subsequent heat treatment causing complete conversion to W^6+^ (Supplementary Fig. S3a). The XPS Al 2 s spectra confirmed that the WO_3_ phase after heat treatment contained no elemental Al (Supplementary Fig. S3b).

**Figure 4:**
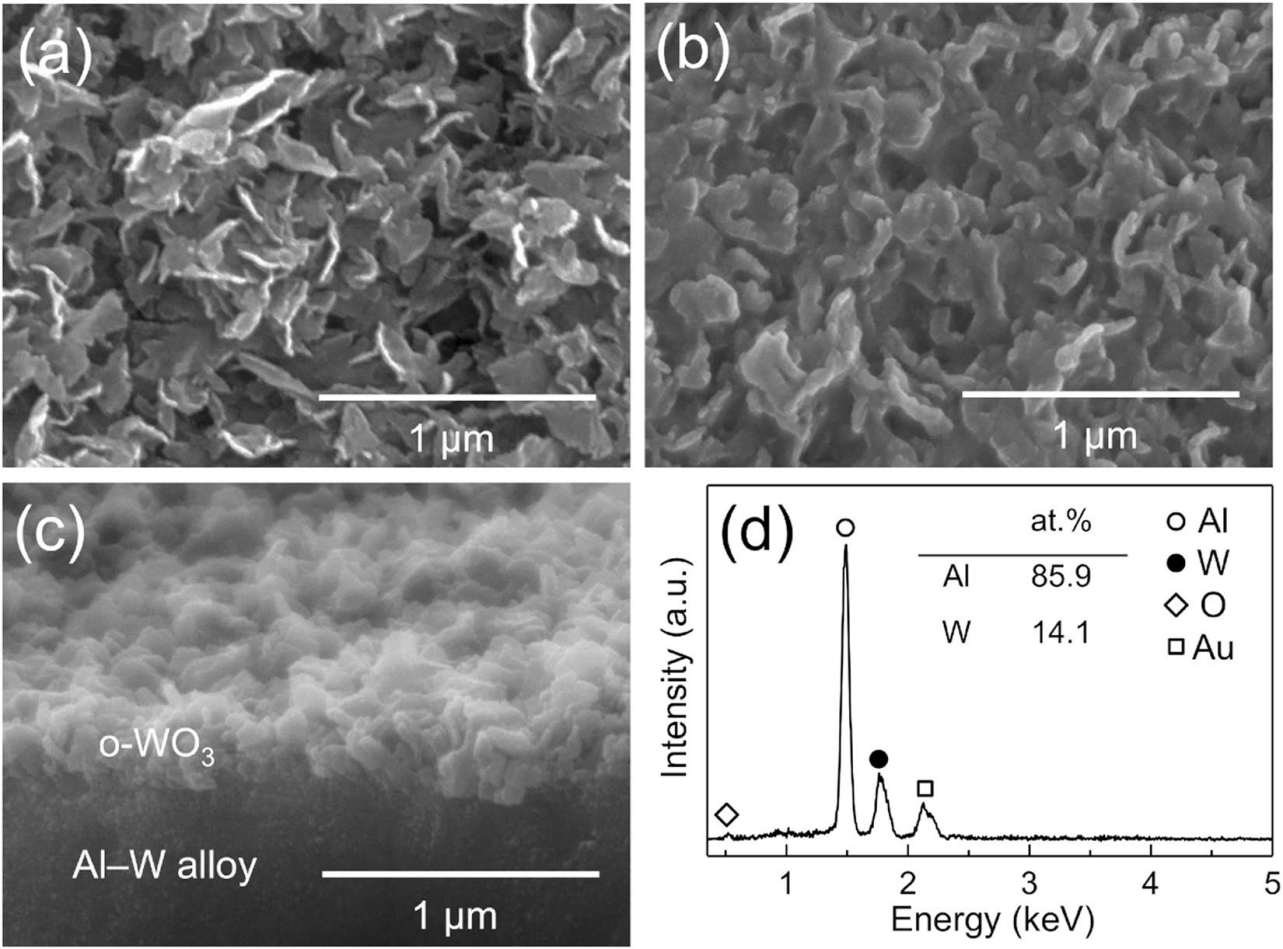
Surface and cross-sectional SEM images of the Al–W alloy films after selective dissolution and heat treatment at (**a**) 300 °C and (**b**,**c**) 350 °C. (**d**) EDX spectrum of the Al–W underlayer.

Surface SEM images (Fig. 4a,b) revealed that the petal-like grains were almost unchanged by heating at 300 °C, whereas the grains were sintered at 350 °C to form more compact grains. The cross-sectional image of an Al–W alloy film following heat treatment at 350 °C (Fig. 4c) shows that an ~300 nm thick WO_3_ layer was formed on the film. The EDX spectrum of the Al–W alloy underlayer (Fig. 4d) indicated that it remained unoxidized, and the alloy composition was nearly identical to that of the as-deposited film.

It was thought that heat treatment at even higher temperatures might generate WO_3_ with enhanced photocatalytic performance^49^. However, heat treatment at temperatures of 400 °C or higher crystallized the amorphous Al–W alloy to form intermetallic compounds such as Al_5_W and Al_12_W (Supplementary Fig. S4). The films containing these compounds were quite brittle and thus impractical for use as coatings.

### Photocatalytic self-cleaning properties of WO_3_/Al–W alloy films

Photocatalytic degradation of SA and methylene blue (MB) on the WO_3_/Al–W alloy films obtained by selective dissolution and heat treatment as described above was examined to evaluate the self-cleaning properties of the films. The photodecomposition of SA was monitored by measuring the change in the water contact angle on the film coated with SA. Absorption spectra of MB solutions in contact with the films were analyzed to monitor the photodegradation of MB.

**Figure 5:**
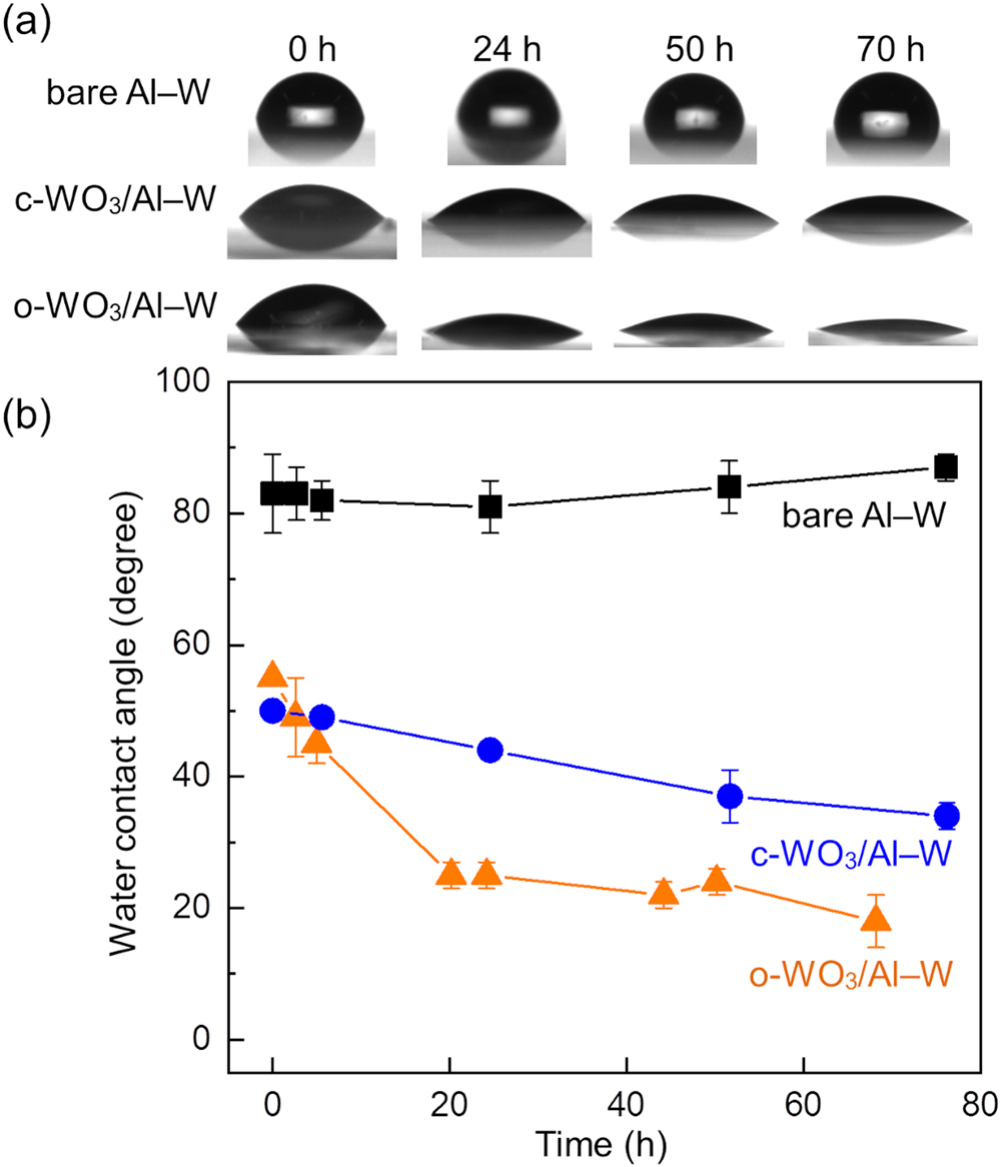
(**a**) Shapes of water droplets and (**b**) water contact angles on bare Al–W, c-WO_3_/Al–W, and o-WO_3_/Al–W alloy films under visible light illumination.

The water contact angle on each film was measured under illumination with visible light after the film was treated with SA solution in heptane. For comparison, the contact angle on a bare Al–W alloy film was also measured following treatment with SA solution in heptane. As shown in Fig. 5, the contact angle on each film prior to illumination (*t* = 0 h) exceeded 50° due to the hydrophobicity of the SA adsorbed on the film surfaces. The contact angle for the bare Al–W alloy films remained almost constant regardless of illumination time, indicating the SA was present on the surface. In contrast, the contact angles on the c-WO_3_ and o-WO_3_ films decreased with increasing illumination time, indicating these films photocatalyzed the degradation of SA. The contact angle decreased more rapidly on the o-WO_3_ film than on the c-WO_3_ film.

**Figure 6:**
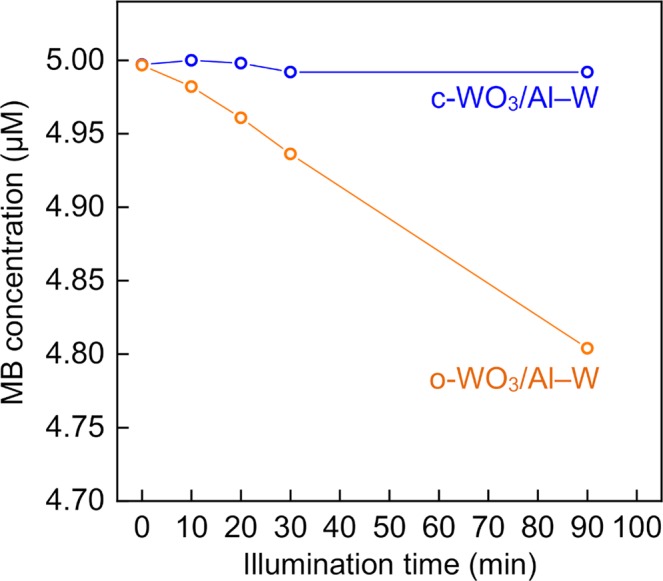
Concentration of MB in aqueous solutions in contact with c-WO_3_/Al–W and o-WO_3_/Al–W alloy films under visible light illumination.

The concentrations of MB in aqueous solutions in contact with the o-WO_3_ and c-WO_3_ films under visible light illumination were calculated from the absorption spectra of MB (Supplementary Fig. S5). The variations of the MB concentration in aqueous solutions in contact with the c-WO_3_ and o-WO_3_ films under visible light illumination are shown in Fig. 6. The MB concentration remained almost constant in the solution with the c-WO_3_ film, whereas the MB concentration decreased with increasing illumination time in the solution with the o-WO_3_ film. This result demonstrated that the o-WO_3_ film could photocatalyze the degradation of MB. The inability of the c-WO_3_ film to photocatalyze the degradation of MB has been reported elsewhere and was attributed to the c-WO_3_ bandgap of 2.0 eV^50^, which is lower than those of WO_3_ in the orthorhombic and monoclinic phases (2.5–2.8 eV)^49,51,52^.

Based on the photocatalytic degradation results for SA and MB, the o-WO_3_/Al–W alloy film photodegraded organic compounds adsorbed on its surface more effectively than the c-WO_3_/Al–W film. Thus, the self-cleaning ability of the o-WO_3_/Al–W alloy film is superior to that of the c-WO_3_/Al–W film.

### Corrosion resistance of the WO_3_/Al–W alloy films

**Figure 7:**
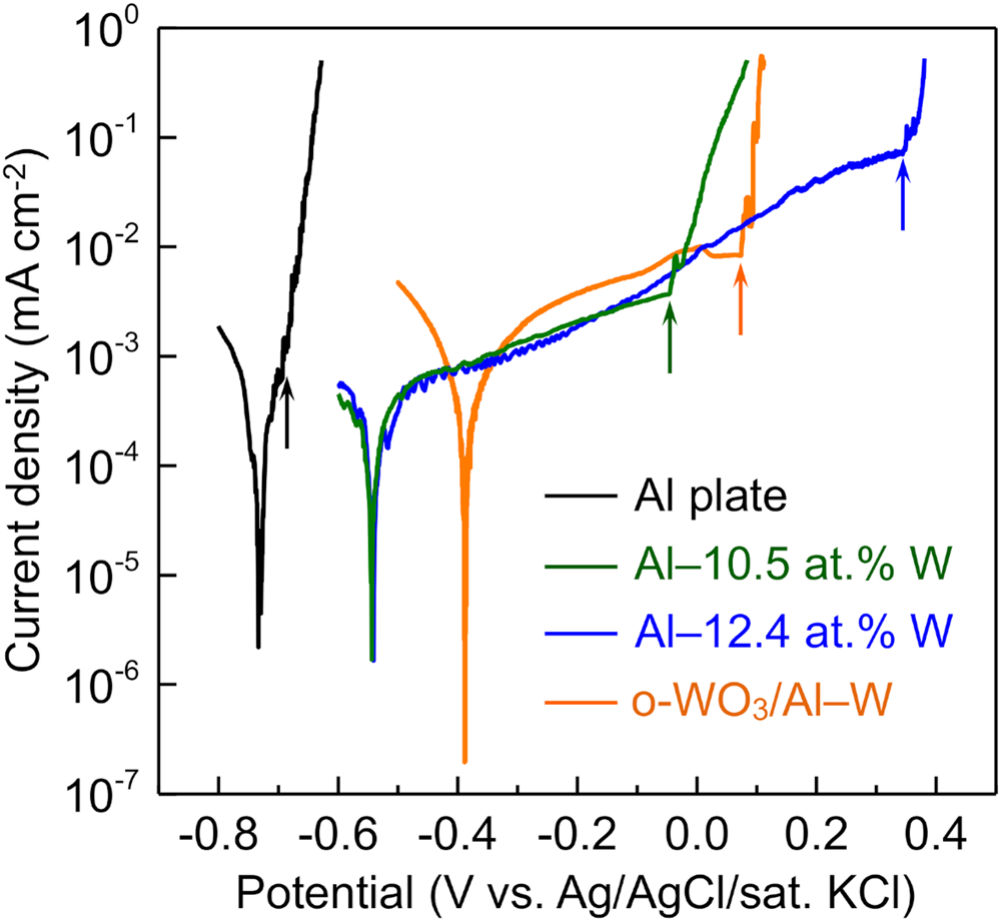
Polarization curves for an o-WO_3_/Al–W alloy film, bare Al–W alloy films with 12.4 and 10.5 at.% W, and an Al plate in 3.5 wt.% aq. NaCl solution. The arrows indicate the pitting potential in each curve.

The resistance of the o-WO_3_/Al–W alloy film to pitting corrosion was investigated by measuring the pitting potential in 3.5 wt.% aqueous NaCl solution through potentiodynamic polarization tests. The polarization curve of the o-WO_3_/Al–W alloy film is shown in Fig. 7, with the polarization curves of bare Al–W alloy films containing 12.4 and 10.5 at.% W and an Al plate included for comparison. In each of the curves, the anodic current density exhibited a steep rise at a certain potential. This steep rise was attributed to pitting corrosion on the surface of the alloy films. The pitting potential of the o-WO_3_/Al–W alloy film was lower than that of the bare alloy film with a similar W content (12.4 at.% W); however, it was higher than those of the Al–W alloy film with 10.5 at.% W and the Al plate.

## Conclusions

A photocatalytic WO_3_ surface layer was formed on electrodeposited Al–W alloy films by selective dissolution and heat treatment. The selective dissolution of Al and oxidation of W proceeded during immersion of the Al–W alloy films in an aqueous HNO_3_ solution and nanostructured H_2_WO_4_·H_2_O was formed on the alloy surface. Subsequently, H_2_WO_4_·H_2_O was dehydrated to c-WO_3_ or o-WO_3_ by heat treatment at 300 or 350 °C, respectively. The orthorhombic WO_3_/Al–W alloy film exhibited superior visible-light photocatalytic activity for the photodegradation of SA and MB adsorbed on the surface. Although the resistance of the WO_3_/Al–W alloy film to pitting corrosion was slightly lower than that of the untreated Al–W alloy film, it was still better than that of the Al–W alloy film with 10.5 at.% W. These results demonstrate the feasibility of selective dissolution and heat treatment as a new process to obtain corrosion-resistant alloy films with photocatalytic self-cleaning abilities under visible light illumination.

## Methods

### Electrodeposition of Al–W alloy films

The electrodeposition of Al–W alloy films was carried out in an EMIC–AlCl_3_–W_6_Cl_12_ bath placed in an argon-filled SDB-1AO glove box (Miwa Manufacturing Co., Japan). An EMIC–AlCl_3_ melt was first prepared by slowly adding AlCl_3_ (99%, Fluka, USA) to EMIC (97%, Tokyo Chemical Industry, Japan) in a molar ratio of 2:1. EMIC was vacuum-dried at 120 °C prior to use. The prepared melt was stored in a 25 mL glass vessel, which served as the electrolytic cell. W_6_Cl_12_, synthesized by a method described elsewhere^17,18^, was added to the melt to a final concentration of 49 mM. The bath temperature was maintained at 80 °C throughout the experiment with a TJA-550 thermostat (ASONE Corp., Japan) connected to a rubber heater wound around the cell and a thermocouple immersed in the bath.

Galvanostatic electrodeposition was performed on a 100 nm thick Cu film formed on a glass substrate by sputtering. A section of the Cu/glass substrate was covered with polytetrafluoroethylene tape, such that a defined 1 × 1 cm^2^ area was exposed to the bath. An Al plate served as the counter electrode. The Cu/glass substrate and Al plate were placed vertically in the cell containing the melt. The Cu/glass substrate and Al plate were parallel to each other, and the distance between them was approximately 10 mm. During electrodeposition, the bath was stirred at 150 rpm with a stirrer bar (15 × 5 mm) and a PC-420D magnetic stirrer (Corning, USA). Electrodeposition was performed at 20 mA cm^−2^ for 25 min, with the current density controlled by a 660 C electrochemical analyzer (ALS Co., Japan).

### Selective dissolution and heat treatment of Al–W alloy films

The electrodeposited Al–W alloy films were mechanically polished to obtain smooth surfaces. The portion of each Cu substrate on which the alloy film was not deposited was covered with KTC-AC-828T masking resin (Kakoki Trading Co., Japan), after which the films were immersed in 0.56 M (3.5 wt.%) aqueous HNO_3_ solution at room temperature.

The films were heat treated in a KBF848N1 electric furnace (Koyo Thermo Systems Co., Japan) in air. The films were heated from room temperature to the desired temperature at a rate of 2 °C min^−1^, held at that temperature for 10 h, and then cooled slowly over several hours to room temperature.

The surface and cross-sectional morphologies of the alloy films were examined by field-emission scanning electron microscopy (FE-SEM), and the elemental compositions of the films were determined by EDX on a SU6600 field-emission scanning electron microscope (Hitachi, Japan) equipped with a Quantax Xflash 4010 detector (Bruker, USA). To prevent charging effects during the SEM analysis, a thin Au coating was deposited onto the samples by sputtering. The crystal structures of the films were determined by XRD analysis using an X’pert PRO-MPD diffractometer (Malvern Panalytical, UK). The XPS spectra of the film surfaces were measured by JPS-9030 (JEOL, Japan) with Mg Kα (1253.6 eV) X-ray source. The spectra in this paper were calibrated using C1s peak at 285.0 eV.

### Evaluation of photocatalytic self-cleaning properties

The photocatalytic self-cleaning properties of the films were evaluated by monitoring the photodegradation of SA and MB^21^. The reagents used in these tests were purchased from Wako Chemical Co. (Japan) and used as received. For both tests, the films were illuminated with visible light at wavelengths from 380 to 520 nm. The light was emitted from a HAL-320 xenon solar simulator lamp (Asahi Spectra, Japan) with a light intensity of 1 sun passed through a Super Cold 750 filter and an SHPF-25C-533 short-pass filter (SIGMA KOKI, Japan) with a transmittance range of 380–520 nm. Wavelengths of >520 nm were blocked to prevent self-decomposition of MB via light absorption.

The photodegradation of SA was monitored according to the Japan Industrial Standards R_1753 method (2013). Each alloy film was illuminated with a LUV-16 UV lamp (ASONE Co., Japan) for 24 h in a dark room to ensure that the surface was devoid of interferents. Each film was then coated with a thin layer of SA by spin-coating 1 mL of 0.3 wt.% SA solution in heptane at 2000 rpm and dried at 70 °C on a hot plate for 10 min. Following SA coating, each film was illuminated under visible light from the solar simulator lamp in a dark room. The contact angle of a water droplet (<1 μL) on each film was measured with a ME2 contact angle meter (Asumi Giken Co., Japan).

For the photodegradation of MB, each film was first immersed in a large volume of aqueous 5 μM MB solution for >12 h to ensure that the adsorption/desorption equilibrium was reached. The film was then placed in a 4.5 mL acryl vial containing 1.5 mL of 5 μM MB solution and illuminated with visible light. The absorption spectrum of the MB solution was collected with a UV-2450 UV-VIS spectrometer (Shimadzu, Japan). The absorbance at 663 nm was used to determine the MB concentration according to Beer’s law.

### Evaluation of corrosion resistance

The resistance of the alloy films to pitting corrosion was evaluated by potentiodynamic polarization in a 3.5 wt.% aqueous NaCl solution. The NaCl solution was deaerated by bubbling with argon gas for 1 h prior to use. The electrochemical measurements were carried out with a three-electrode system using a 25 mL glass vessel as an electrolytic cell. A section of each alloy film was covered with a masking resin to expose a defined 0.2 mm^2^ area that served as the working electrode. A platinum plate and a Ag/AgCl/saturated KCl electrode were used as the counter and reference electrodes, respectively. The potential was scanned in the positive direction from −800 mV vs. Ag/AgCl/sat. KCl at a rate of 0.5 mV s^−1^.

## Supplementary information

Supplementary information

## Data Availability

The datasets generated and analyzed during the present study are available from the corresponding author on reasonable request.
