# Supplementary material for: Formation of a photocatalytic WO3 surface layer on electrodeposited Al–W alloy coatings by selective dissolution and heat treatment
Source: Sci Rep. 2019 Nov 5;9:16008. doi: 10.1038/s41598-019-52178-6 (PMC6831697; doi:10.1038/s41598-019-52178-6)
Supplement: Supplementary file 1 — Supplementary information [file 41598_2019_52178_MOESM1_ESM.pdf]

Supplementary materials for

Formation of a photocatalytic WO_3 surface layer on electrodeposited Al–W alloy coatings by selective dissolution and heat treatment

Shota Higashino[1], Masao Miyake[1, *], Takumi Ikenoue[1], and Tetsuji Hirato[1]

[1]Graduate School of Energy Science, Kyoto University, Yoshida-honmachi, Sakyo-ku, Kyoto 606-8501, Japan

*Corresponding author
TEL: +81-75-753-5914, E-mail: miyake.masao.4e@kyoto-u.ac.jp

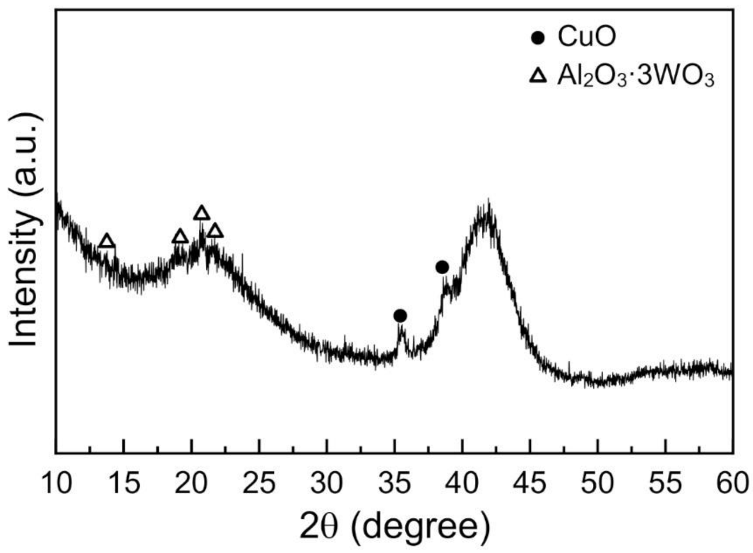

Supplementary Figure S1 (a) XRD pattern of the Al–W alloy film after heat treatment at 350 °C for 10 h. The diffraction pattern of CuO is derived from the oxidation layer of the Cu substrate outside the electrodeposition area.

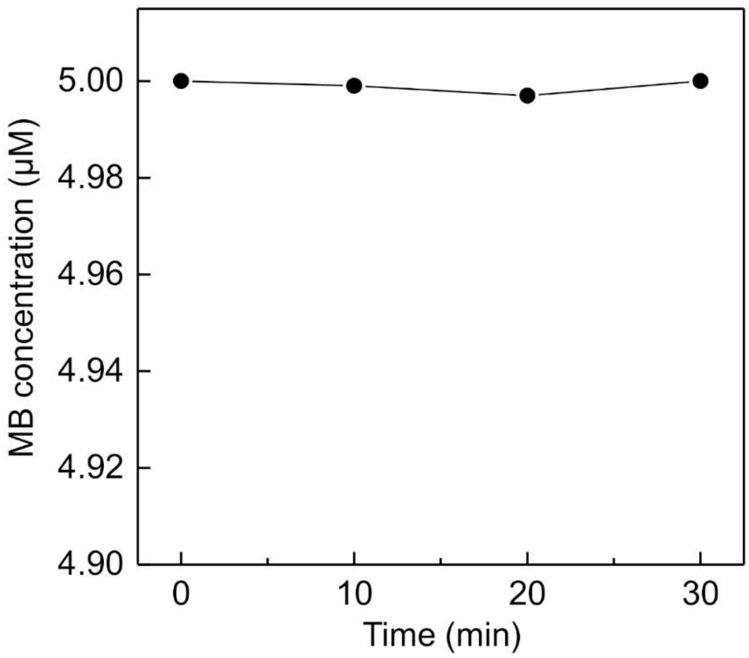

Supplementary Figure S2 Concentration of MB in aqueous solution in contact with the Al–W alloy film after heat treatment at 350 °C under visible light illumination.

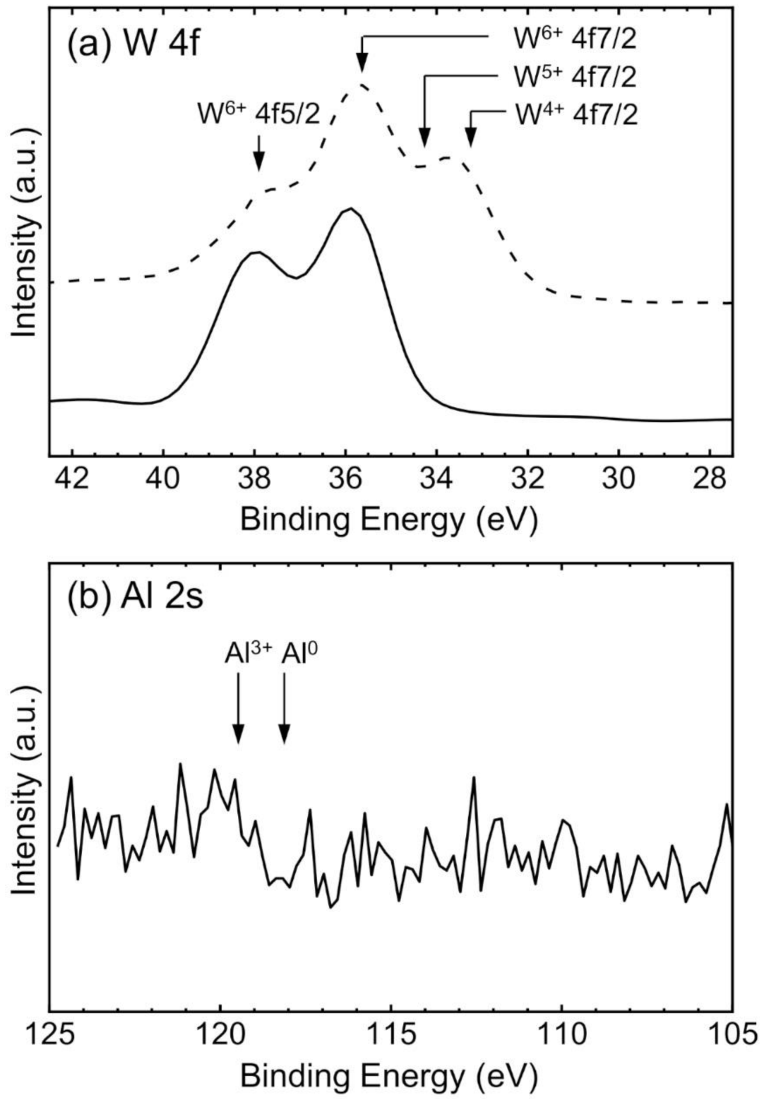

Supplementary Figure S3 (a) W 4f XPS spectra of Al–W alloy films after selective dissolution for 15 h (dashed line) and after selective dissolution and heat treatment at 350 °C (solid line). (b) Al 2s XPS spectra of an Al–W alloy film after selective dissolution and heat treatment at 350 °C. The binding energies of W^{4+}, W^{5+}, W^{6+}, Al^0, and Al^{3+} are denoted by arrows.

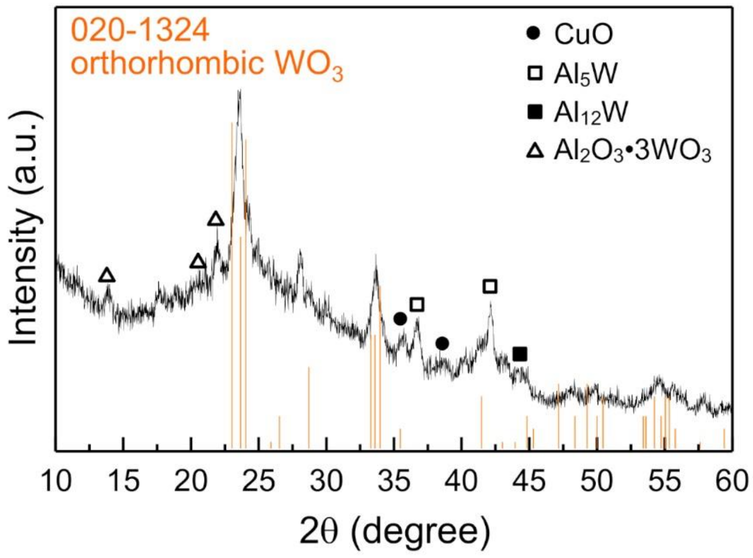

Supplementary Figure S4 XRD pattern of an Al–W alloy film after selective dissolution for 15 h and heat treatment at 400 °C. The diffraction pattern of aluminum tungsten oxide ($Al_2O_3 \cdot 3WO_3$) is attributable to oxidation of the Al–W alloy underlayer.

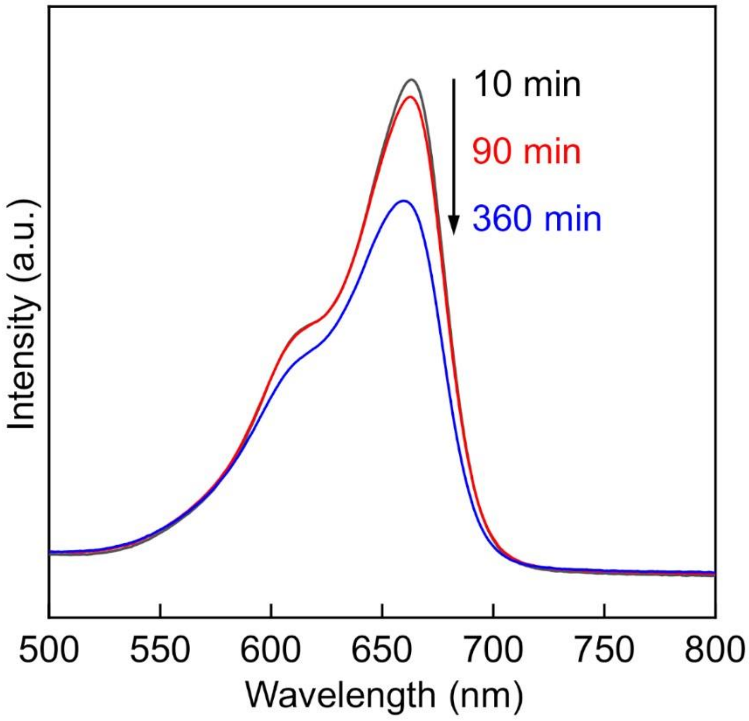

Supplementary Figure S5 Absorption spectra of a MB aqueous solution in contact with the o-WO_3/Al–W alloy film under visible light illumination.
